# Laparoscopic Excision of a Large Intra-abdominal Cystic Lymphangioma in a Six-Month-Old Infant

**DOI:** 10.7759/cureus.76427

**Published:** 2024-12-26

**Authors:** Theodoros Spinos, Elisavet Kanna, Zoi Lamprinou, Irene Yerolemidou, Ioannis Skondras

**Affiliations:** 1 Urology, University Hospital of Patras, Patras, GRC; 2 Pediatric Surgery, Panagiotis and Aglaia Kyriakou Children's Hospital, Athens, GRC

**Keywords:** children, cystic lymphangioma, laparoscopy in infants, lymphatic malformation, minimally invasive surgical procedures

## Abstract

Lymphatic malformation is a rare vascular anomaly caused by abnormal lymphatic system development during embryogenesis. Intra-abdominal lymphatic malformations are uncommon in children, and surgical excision is considered the gold standard for treatment. However, few reports of minimally invasive laparoscopic approaches have been documented. This case report presents a six-month-old infant with a large intra-abdominal cystic lymphangioma, diagnosed prenatally and treated laparoscopically. The lesion was excised en bloc using low-impact laparoscopy, with an uneventful intraoperative and postoperative course. This case highlights the feasibility and safety of laparoscopic excision in young patients, even for large masses. Further studies are needed to establish the role of minimally invasive surgery in pediatric patients with large intra-abdominal lymphatic malformations.

## Introduction

Lymphatic malformation is a vascular abnormality, which is caused by anomalous development of the lymphatic system during embryogenesis [1]. Intra-abdominal lymphatic malformations are not common in children, while their gold standard treatment is considered the surgical excision of the lesion [2,3]. Only a few cases of a minimally invasive approach for their excision have been described in the literature [1-5]. The purpose of this case report is to present the case of a six-month-old infant with a large cystic intra-abdominal lymphangioma, who was treated laparoscopically in our department.

## Case presentation

A six-month-old full-term infant was referred to our hospital due to a prenatally detected large cystic intra-abdominal mass for further investigation and treatment. Written consent was obtained from the parents of the infant. The antenatal ultrasound, performed at 36 weeks and five days of pregnancy, revealed an intra-abdominal cystic formation measuring 51×48×25 mm, with relatively thin walls and of unknown origin. Following these findings, the newborn was hospitalized immediately after an uncomplicated labor in the neonatal intensive care unit (NICU) for two days. A postnatal ultrasound during NICU hospitalization confirmed the presence of a thin-walled cystic mass (8.48x3.57 cm) in the right pararenal space, extending beyond the midline to the left. Multiple echogenic elements were found within the mass, while no vasculature was detected. At that point, the diagnosis of dysplasia of lymphatic origin was prioritized among the differential diagnoses. Other potential differential diagnoses included mesenteric cyst, enteric duplication cyst, adrenal hemorrhage, neuroblastoma (cystic variant), and renal cystic disease (e.g., multicystic dysplastic kidney or hydronephrosis). Further imaging and diagnostic workups were planned to confirm the nature of the mass and guide appropriate management.

**Figure 1:**
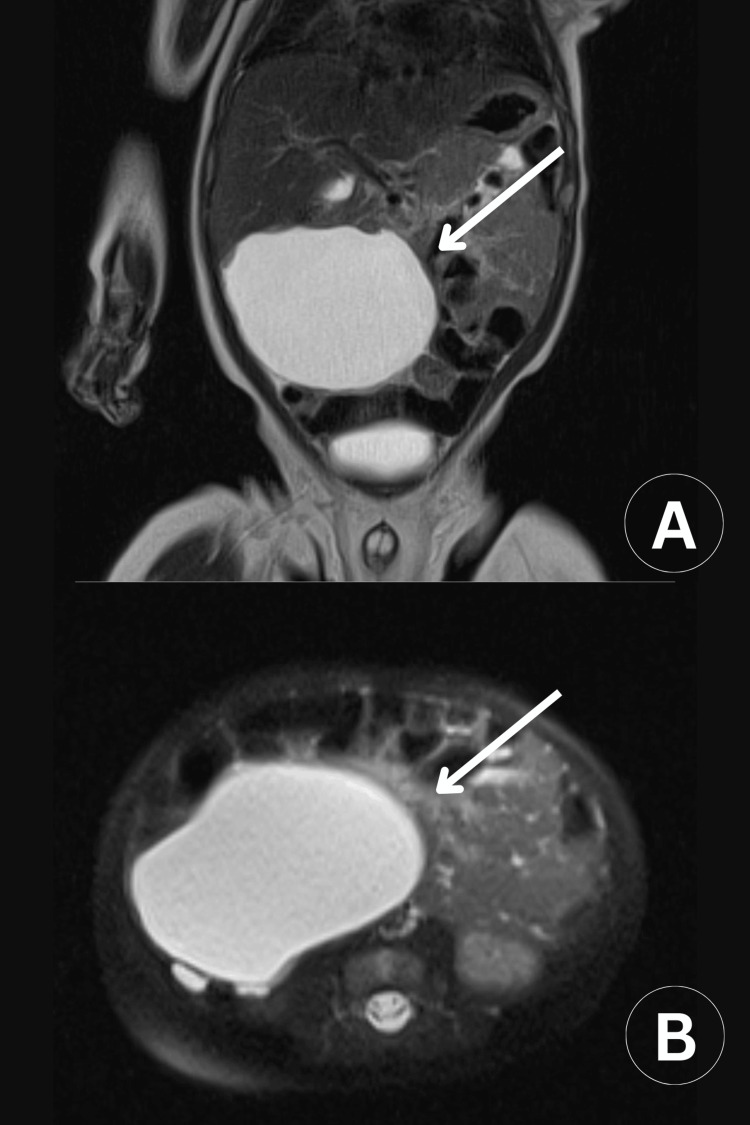
MRI of a large, well-defined cystic lymphangioma. (A) Coronal view with high T2 signal intensity. (B) Axial view with high T2 signal intensity.

A third ultrasound was performed at the age of two months at our hospital. This revealed a large thin-walled cystic mass measuring 7.61x3.58x5.47 cm, with a volume of approximately 78 cc. The mass occupied the central and lower abdomen, extending cephalocaudally from the level below the pancreas to the upper border of the bladder on either side of the midline. A large main cystic space and multiple smaller compartments, separated by septa, were identified. Doppler ultrasound did not reveal any internal or peripheral vasculature. Posteriorly, the mass extended into the prevertebral space, coming into contact with underlying vascular structures without infiltrating them. These findings suggested the presence of a cystic lymphangioma. An MRI scan described a large cystic subhepatic lesion in the right side of the abdomen, located anterior to the kidney, psoas muscle, aorta, and thoracic duct, with dimensions of 5.5x6x7.6 cm (Figure 1). A final ultrasound was performed before surgery, showing a large thin-walled cystic mass measuring 6.92x5.81x6.41 cm, with a volume of approximately 134 cc. Repeated ultrasounds were conducted to closely monitor the lesion’s size, volume, and structural characteristics over time. This repeated imaging was essential for assessing potential changes in the lesion, understanding its relationship to surrounding anatomical structures, and ensuring accurate and safe preoperative planning.

**Figure 2:**
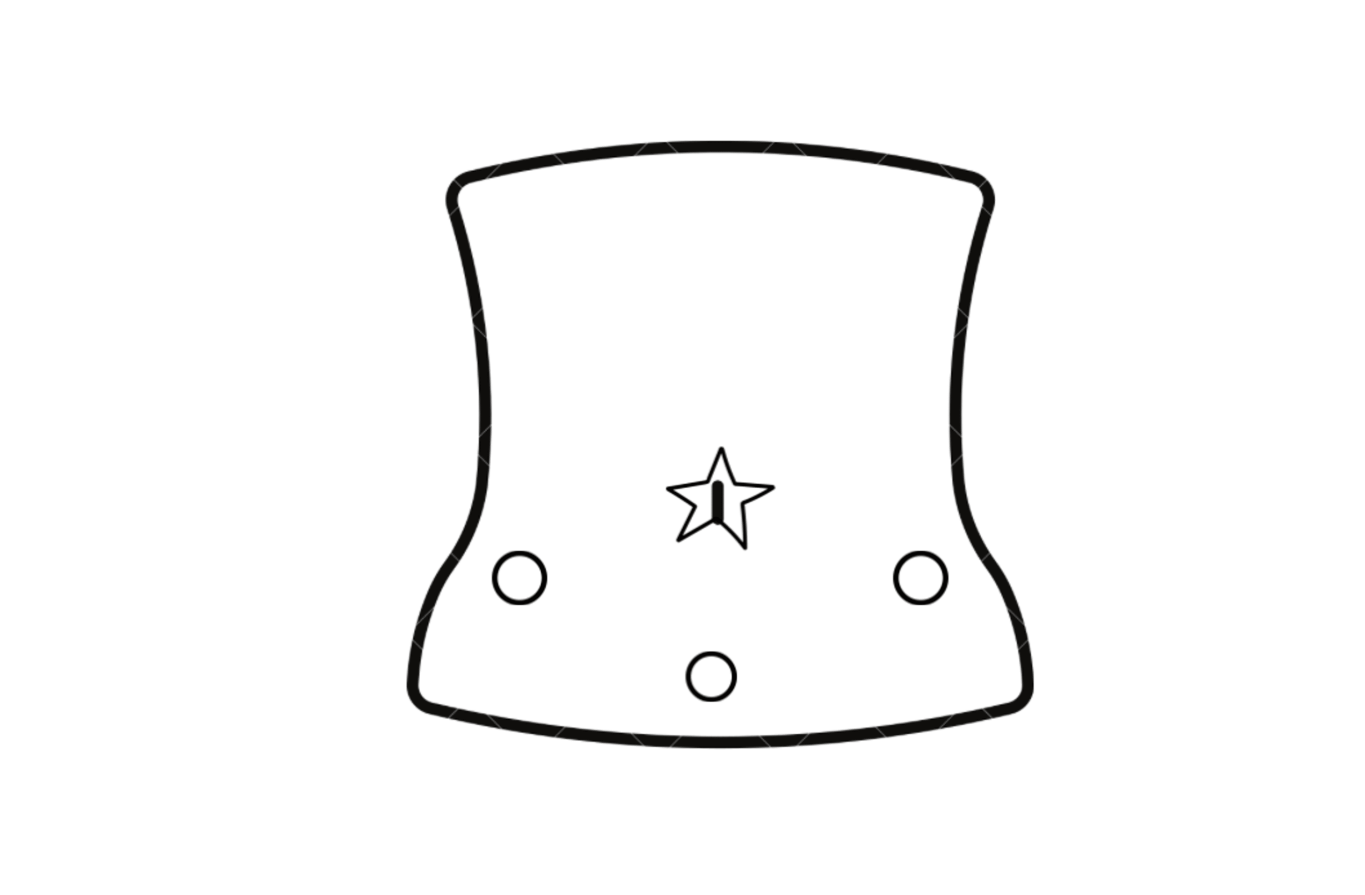
The image depicts the placement of trocars in an infant’s abdomen for a laparoscopic procedure: umbilical (star), hypogastric, and right and left lumbar regions.

**Figure 3:**
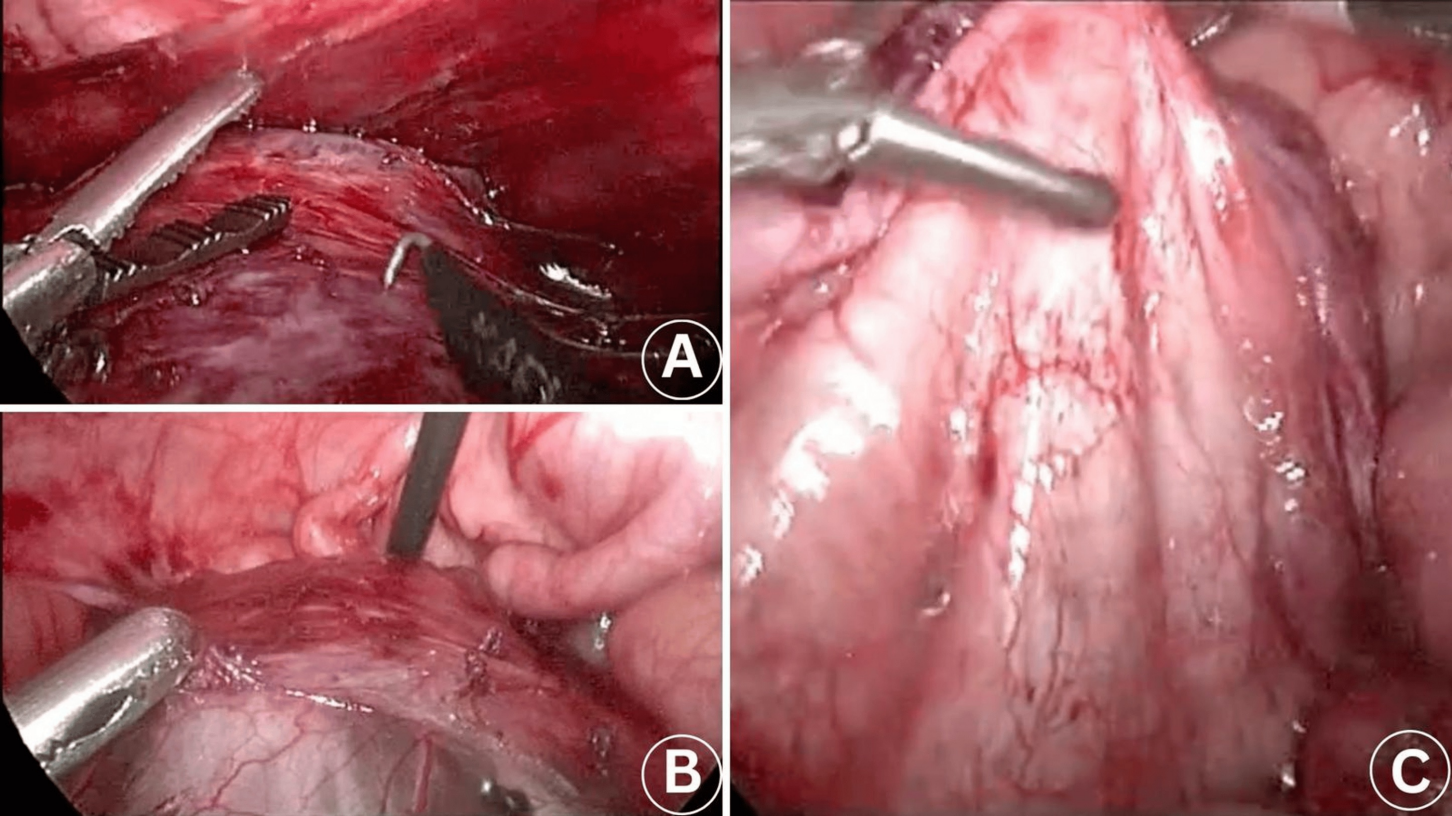
Intraoperative view of a large intra-abdominal cystic lymphangioma. (A,B) The lesion was precisely dissected and carefully detached from the surrounding tissues, minimizing disruption to adjacent structures. (C) The lesion following complete laparoscopic excision.

The procedure was performed under low-impact laparoscopy conditions, with the pneumoperitoneum remaining stable at 6 mmHg throughout the entire procedure, with the aid of the AirSeal® System. Total operative time was 150 minutes, while hemoglobin drop was only 1 mg/dL. An incision through the umbilicus was performed, and the optical trocar was inserted into the abdomen, while the pneumoperitoneum was created with the Hasson technique. Using a 5 mm 30° scope, another three 3 mm trocars were placed: one in the hypogastrium, one in the right lumbar region, and one in the left lumbar region (Figure 2). A large mass posteriorly to the ascending colon was recognized, extending toward the midline and being located retroperitoneally. The lesion was carefully dissected and meticulously separated from the surrounding tissues. It did not extend in close proximity to the kidney or ureter but was in close proximity to the mesenteric vessels. It was decompressed using an orange Abbocath 14G, which revealed thick, yellow-colored fluid. The lesion was then excised en bloc and removed through the umbilicus using a retrieval bag (Figure 3). The abdomen was deflated, and the trocars were removed under direct vision. The pathologist’s report confirmed that the histological findings were consistent with a cystic lymphangioma, ruling out a mesenteric cyst. The patient's intraoperative and postoperative course was uneventful, and he was discharged from the hospital on the second postoperative day.

## Discussion

Only a few cases of laparoscopic- or robotic-assisted excisions of cystic lymphangiomas in children have been described in the literature. Singh et al. reported a complete surgical excision of a retroperitoneal cystic lymphangioma via laparoscopy in a 15-year-old girl [1]. Liu et al. also shared their outcomes regarding laparoscopic resection of intra-abdominal lymphatic malformations in 10 children, concluding that laparoscopy is a safe and effective option for such cases in the pediatric population [2]. Solari et al. described one case of laparoscopic excision of a retroperitoneal lymphatic malformation in a newborn, reporting that neonatal laparoscopy can achieve both diagnosis and management of prenatally suspected abdominal lymphatic malformations [3]. Lagausie et al. reported their experience with the laparoscopic approach to lymphangiomas, examining 15 cases of lymphangiomas in children aged five months to 14 years, all of which were treated laparoscopically [4]. Finally, Wildhaber et al. reported two cases of laparoscopic excision of retroperitoneal cystic lymphangiomas in an 18-month-old girl and a 4-year-old boy, respectively, concluding that complete laparoscopic excision should be considered a therapeutic strategy for treating retroperitoneal cystic lymphangiomas in children [5].

From an oncological perspective, laparoscopy can be a safe option in such cases, provided that specific considerations are taken into account during surgery. These include ensuring complete excision of the mass with clear margins to minimize the risk of recurrence, careful handling to avoid rupture or spillage of cystic contents, and thorough exploration of the abdominal cavity for additional lesions [6]. In our case, the peculiarity was that the patient was at a very young age (six months old), while the intra-abdominal mass was of large dimensions, rendering the laparoscopic excision extremely challenging. Laparoscopy in such scenarios requires advanced surgical expertise, appropriate preoperative imaging to map the anatomy, and intraoperative tools to ensure both precision and safety.

Sclerotherapy is a non-surgical treatment alternative for lymphatic malformations, including abdominal lymphangiomas, particularly in cases where surgery poses significant risks or is deemed unsuitable. This approach involves injecting sclerosant agents, such as doxycycline or bleomycin, directly into the cystic spaces to induce fibrosis and shrinkage of the lesion [7,8]. While sclerotherapy has shown promising results in reducing the size of lymphangiomas, its efficacy in completely resolving larger or multiloculated lesions, such as the one described in this case, remains variable. Additionally, potential complications, including infection, localized inflammation, or incomplete resolution, may require further interventions. For large or symptomatic abdominal lymphangiomas, especially those affecting vital structures, sclerotherapy may serve as an adjunct or alternative to surgery, though it is generally less favored in comparison to complete laparoscopic excision, which ensures definitive treatment. Future studies comparing long-term outcomes of sclerotherapy and minimally invasive surgery in pediatric lymphangiomas are necessary to optimize treatment strategies.

## Conclusions

Laparoscopic excision of large intra-abdominal cystic lymphangiomas in children is a feasible, safe, and efficient approach in high-volume laparoscopic departments when performed by experienced surgeons. The advantages associated with minimally invasive procedures, such as less blood loss, shorter hospital stays, and faster recovery, are also applicable to this approach. However, further studies documenting laparoscopic excision of large intra-abdominal masses in children are needed to draw broader and safer conclusions. 
